# An industry perspective on current QSP trends in drug development

**DOI:** 10.1007/s10928-024-09905-y

**Published:** 2024-03-05

**Authors:** Lourdes Cucurull-Sanchez

**Affiliations:** Clinical Pharmacology Modelling and Simulation, GlaxoSmithKline, Stevenage, Hertfordshire UK

**Keywords:** Quantitative systems pharmacology, QSP, MIDD, MID3

## Abstract

2023 marks the 10th anniversary of Natpara’s submission to the US FDA, which led to the first recorded regulatory interaction where a decision was supported by Quantitative and Systems Pharmacology (QSP) simulations. It had taken about 5 years for the timid QSP discipline to emerge as an effective Model-Informed Drug Development (MIDD) tool with visible impact in the pharmaceutical industry. Since then, the presence of QSP in the regulatory environment has continued to increase, to the point that the Agency reported 60 QSP submissions in 2020 alone, representing ~ 4% of their annual IND submissions [1]. What sort of industry mindset has enabled QSP to reach this level of success? How does QSP fit within the MIDD paradigm? Does QSP mean the same to Discovery and to Clinical Development projects? How do ‘platforms’ compare to ‘fit-for-purpose’ QSP models in an industrial setting? Can QSP and empirical Pharmacokinetic-Pharmacodynamic (PKPD) modelling be complementary? What level of validation is required to inform drug development decisions? This article reflects on all these questions, in particular addressing those audiences with limited line-of-sight into the drug industry decision-making machinery.

## Introduction

**Fig. 1:**
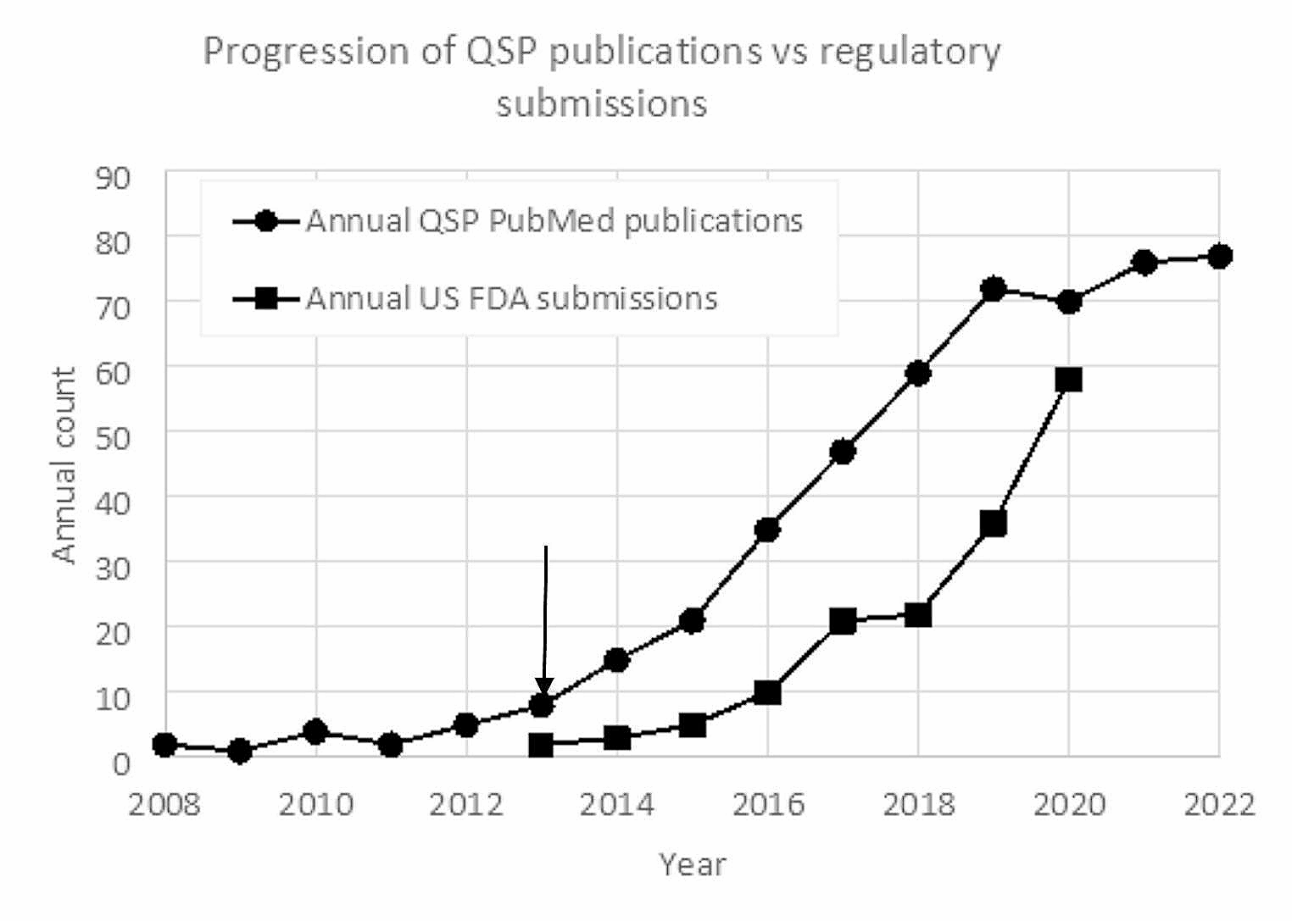
Comparison of the progression in the number of annual QSP publications (according to PubMed, updated from [13]) vs. regulatory submissions reported by the US FDA [11, 12]. The arrow marks the time of the first recorded QSP-based regulatory submission. If the growth in publications is indeed approaching a plateau (see text), and the annual growth rates of regulatory submissions stayed the same since 2020, then the QSP activity and impact in late stages of industry has potentially reached the public domain levels

This year marks the 10th Anniversary of the regulatory approval of the so-called ‘Natpara case’ – the first reported instance where Quantitative Systems Pharmacology influenced a regulatory decision. The FDA Office of Clinical Pharmacology used a calcium homeostasis QSP model to support their request for a post-marketing clinical trial to explore how alternative dosing strategies could reduce an adverse event [2]. To QSP practitioners, that was a key milestone (‘a watershed moment?’) [2], a breath of fresh air since the coinage of the QSP concept in 2008 [3, 4] because it illustrated the potential that QSP had to help the pharmaceutical industry in its drug development process, and proved that QSP was certainly not a ‘flash in the pan’ [5]. From that moment on, the critical mass of QSP within the modelling and simulations community has kept growing. Its presence in peer-reviewed journals seems to have approached a ‘steady state’ only during the last three years, arguably due to the impact of the Covid-19 pandemic on collaborative efforts (see Fig. 1). QSP has not only become a natural and familiar term in international conferences and workshops, but it has even taken central stage. A great example would be the ‘International Symposium on Measurement and Kinetics of In Vivo Drug Effects’, celebrated in The Netherlands every 4 years since 1990, which has been recently renamed to ‘Quantitative Systems Pharmacology Conference’ [6]. Testament to this is also the increased presence of QSP in professional associations (QSP SIG in ISoP [7], Systems Pharmacology Community in ASCPT’s Quantitative Pharmacology WG [8], QSP Working Groups in the IQ Consortium [9], UK QSP Network [10], etc.) and the significant appearance of new, profitable CROs or business divisions providing QSP consultancy to Pharma Industry [1]. Perhaps the key performance indicator (KPI) that best reflects the degree of impact that QSP has had and continues to have on the industry decision-making process is the overwhelming growth in the number of QSP-based submissions to the US FDA – according to published data [11, 12] these tend to double every ~ 1.4 years (see Fig. 1). Regardless of the eventual success of those submissions, it is clear that the most innovative sectors in industry have adopted QSP as a decision-making tool. But what does this mean for the day-to-day development and application of QSP in industry? What sort of mindset has enabled QSP to reach this level of success? This article intends to address and reflect on all these questions, in particular revealing what has happened behind the scenes to those audiences external to the Big Pharma environment.

### QSP and the MIDD/MID3 paradigm

The MIDD paradigm refers to an industry and regulatory framework designed to enable the integration of experimental and simulated data from the pharmacology, statistics, and biology domains into decision-making processes [14]. Model-Informed Drug Discovery and Development (MID3) is an alternative term used when drug Discovery activities are included in this framework, as opposed to just Clinical Development [15–17].

QSP has slowly but steadily found its place within the MIDD/MID3 suite of pharmacometric methods, which include pharmacokinetic/pharmacodynamic (PKPD), physiologically-based pharmacokinetic (PBPK), population pharmacokinetic (popPK) and disease progression models, or dose/exposure-response analyses (D/E-R). As Fig. 1 shows, the first tangible and publicly visible instance of QSP impact in drug development was recorded in 2013, corresponding to the Natpara submission [2]. A steady annual number of QSP publications had already been present in the literature since 2008, but at this point it started to grow year after year. Possibly by causation, likely by correlation, and certainly not by coincidence, both the scientific (academic or industrial) and the regulatory (agency or industrial) domains began to unravel the value of this new modelling and simulation tool called ‘QSP’. In addition, the percolation of QSP into the regulatory domain was a sign that it was making a dent on the internal decision-making within industry. The initial progression in the literature was faster than in regulatory use – something probably to be expected considering the larger complexity of reaching a regulatory milestone in comparison to peer-reviewed journal publication. However, 6 years down the line an inflection point appeared, where the annual growth of QSP literature publications suddenly came to a stand-still. Possibly, this stagnation reflected industry’s protective attitude towards the competitive intelligence captured within their QSP models, or a saturation of its internal resources (including a those required to put together publications after projects terminations). Arguably this could also be a knock-on effect of the Covid-19 pandemic in 2019, when lock-down measures forced a reduction in opportunities for cross-fertilization between research teams, there was a general shift in scientific focus towards the generation of Covid-centric data, and possibly a broad reduction in resources and productivity. If this is the case, then a new peak should appear as soon as resources return to pre-pandemic levels and the publication backlog can be dealt with. In addition, between 2018 and 2019 the annual growth rate of regulatory submissions equaled that of publications, and it continued to accelerate to the extreme where the number of submissions in 2020 (58) was only 17% below the number of publications (70). If beyond 2020 the annual growth rates of both domains stayed at the same levels as in 2019, then it is likely that the number of submissions is currently the same as or superior to the number of publications. This would be a marker that QSP activity and impact in late stages of industry drug development has caught up with the QSP activity in the general public domain.

This progression of QSP has faced and continues to face the same challenges as any other MIDD/MID3 tools [16], making the implementation of QSP strategies inconsistent across pharma industry and regulatory environments. The challenges could be summarized as follows:

- Lack of awareness or understanding among senior leadership of the return on investment (RoI) that QSP can bring to their organizations by de-risking decisions, accelerating timelines or saving costs.
- ‘Confirmatory mind-set’ of clinical teams, who rely mainly on empirical evidence to address clinically important questions – this relegates QSP approaches to the grade of secondary or exploratory analyses (‘nice-to-have’).
- Rigid operational and cultural expectations around the design of Clinical Development Plans (CDPs) and studies, which limit the transfer of data ownership to QSP practitioners and impose timelines that make the iterative process of modelling difficult.
- Lack of standard qualification requirements or guidelines for QSP-based regulatory report submissions, leading to inconsistency in the format and detail of these.

A couple of recent regulatory milestones may provide QSP with renovated energy to overcome these challenges. In 2022 the FDA converted its MIDD Paired Meeting Pilot Program [18, 19] into a permanent meeting program [20], and recently the International Council for Harmonization of Technical Requirements for Pharmaceuticals for Human Use (ICH) approved the development of guideline M15 to outline MIDD principles with respect to regional regulations and legal frameworks [21]. Given the high complexity of QSP models, the FDA paired meetings may become the optimal forum to dissect and discuss in detail the suitability of QSP simulations for each specific context. The ICH M15 guidelines could help to establish standard practice and qualification requirements for QSP across industry and territories. Both fora could become the engines that QSP needs to raise awareness of its unique value as an MIDD/MID3 tool.

The impact that QSP can achieve within industry is directly proportional to the number and relevance of the decisions it can influence. This would be applicable to any MIDD/MID3 simulation and analysis methodology, perhaps to the extent that this framework could be renamed ‘MID4’, for ‘Model-Informed Drug Discovery and Development Decision-making’. Whilst, conceptually, a QSP model could evolve from end-to-end and benefit all stages of a drug development program, its ability to inform decisions can be very different depending on whether these belong to the Discovery or to the Clinical Development domain. The next section covers the differences between them.

### QSP in Discovery vs. Clinical Development

The drug development pipeline is split into two major phases: Discovery (also referred to as ‘Research’) and Clinical Development (often just termed ‘Development’). The hinge between them is formed by two consecutive milestones: the selection of the optimal molecular entity to be tested in the clinic (CS, Candidate Selection), and the moment when that drug candidate is dosed in a human for the first time ever (FTIH, First Time In Human study).

The concept of QSP was born from the need of a tool that enabled the successful ‘translation of preclinical discoveries into meaningful medical progress’ [3]. Naturally, it was first introduced in industry to support Discovery programs, by projecting efficacy estimates in humans before taking any candidate into the clinic or running any in vivo experiments [3]. Therefore, the weight of QSP within industry has historically been shifted towards Discovery rather than Clinical Development, making Discovery the most prolific area for QSP impact, as confirmed by recent surveys [1, 22]. The key, unique application for QSP in drug Discovery (i.e., pre-CS) is to integrate all the emerging evidence that underpins the therapeutic hypothesis for a specific drug-target-indication triad. Therefore, its main impact on decision-making is to provide a direction of travel with clinical line-of-sight, and to provide focus towards those activities that align with the desired profile for the candidate-selected molecule.

Given the lack of clinical data during the Discovery phases, QSP models in a Discovery setting make estimations of the expected average clinical behaviors resulting from engaging the target with potential drug candidate molecules. Those average population responses can then inform decisions around prioritization between different targets, chemical entities, modalities, or routes of administration, or around the design of in vivo or in vitro functional assays. They can also be used to propose hypotheses on mechanistic biomarkers, or to obtain rough estimates of the therapeutic dose in humans, both of which can provide the initial substrate to inform Clinical Development phase decisions. Examples of more detailed questions and decisions that QSP can inform during Discovery phases have been vastly described elsewhere [22, 23].

At the other side of the CS milestone, the challenge is to understand how the average patient behavior translates to whole patient populations of increasing size along the Clinical Development pipeline. The unique value that QSP brings to programs in Development phases is to hone the variability in patient responses by using the mechanistic knowledge that underpins the therapeutic hypothesis. In a way, QSP could be understood as a method to enrich existing clinical datasets with data and knowledge derived from non-clinical experiments. With this in mind, three major areas of application in clinical Drug Development could be defined, where QSP presents an advantage over more traditional MIDD/MID3 methods:

- **Patient stratification**: By generating simulations of virtual patient populations (see section ‘Fit-for-purpose vs. platform models’), QSP enables the optimization of patient stratification plans on the basis of pharmacodynamic biomarkers and clinical endpoints, instead of cruder physiological or demographic covariates. This aspect of QSP modelling becomes even more powerful when considered in combination with Machine Learning (ML) approaches. Taken to the extreme, and with enough biomarker information at the start of treatment, this simulation of virtual patients could derive into the generation of ‘virtual twins’ [24] or ‘avatars’ for patients enrolled in ongoing clinical studies, which would have the potential to support adaptive clinical study designs and would represent the closest possible approach to truly personalized medicine.
- **Combination therapies**: QSP models describe therapeutic interventions at the cellular level, linking the target molecule to pharmacodynamic (PD) biomarkers through a network of biological events. This makes it possible to simulate drug effects against multiple targets within a single model. The advantage of QSP models is that even when the drug effect is additive at the cellular level, synergistic effects can emerge at the system (patient) level. Given all the possible permutations of doses, dosing regimens and schedules of double and triple drug administrations, QSP simulations can help prioritize the drug combinations to take to the clinic. In addition, this approach can also be used to evaluate a company asset differentiation from standard of care or competitor therapeutics.
- **Novel drug therapies**: This refers to both novel drug modalities and to unprecedented targets/mechanisms. Some novel drug modalities are hard to model using empirical PKPD methods because the therapeutic agent experiences modifications driven by its own chemical or biological properties after dosing – this affects for example cell therapies, oncolytic viruses, ADCs (Antibody-Drug Conjugates) and some vaccines. QSP methods are capable of integrating the biological life-cycle of the therapeutic agent within the PK and PD context of the therapy. In some instances the in-vivo evidence supporting the therapeutic hypothesis at the Development stage is limited or (in the case of in-licensed assets) unavailable. An accelerated QSP approach, utilizing the insights from human genetics analyses, and based on in-vitro and patient’s physiological information, can help increase confidence in the therapeutic hypothesis.

The types of decisions that QSP can inform during clinical Drug Development are common to other MID3 approaches, with some added value in the areas outlined above these lines. They could be grouped in three major categories:

- **Design of study protocols and clinical development plans (CDPs)**: These affect a number of internal decisions and have the potential to affect also regulatory decisions:
  - **Dose and schedule selection**. This is the most common contribution from QSP simulations to Clinical Development, and especially important for drug combinations and bridging between disease indications or patient subpopulations (e.g., pediatric, geriatric, metabolically impaired, etc.). An area of major impact could be Oncology, where the recently launched FDA Project Optimus [25] expects pharmaceutical companies to consider the full dose-exposure-response relationship in addition to the Maximal Tolerated Dose (MTD) in order to optimize approved doses.
  - **PD biomarkers selection and sampling times**. Helping teams identify biomarkers of response and/or their optimal sampling times to test in the clinic could lead to early diagnoses in patients, ultimately accelerating the execution of the clinical development program.
  - **Patient inclusion/exclusion criteria for clinical studies.** Putting forward to teams mechanistic (instead of demographic) baseline disease characteristics that are strongly associated with response could lead to highly efficient patient inclusion/exclusion criteria. This needs to be balanced with the operational requirements of the patient enrolment process, such as sufficient prevalence of the patient subpopulation selected, or the development of robust a companion diagnostic [26].
  - **Go/No-go decisions in adaptive studies or gated designs.** Early estimates of the average and distribution of the expected level of response across patients can inform the probability of success (PoS) and go/no-go criteria of a planned study when integrated with statistical Quantitative Decision-Making methods [27].

- **Prioritization of investment areas**: These are decisions exclusively within the pharma company domain. QSP can provide quantitative evidence to inform:
  - **Selection of drug combinations**. It would be practically impossible to test all the possible asset combinations in the clinic, so QSP can provide estimates of the expected patient population responses to doublet or triplet therapies, to help prioritize those from the point of view of pharmacological effect. QSP models developed to this end can eventually add further value for the dose and schedule selection in CDPs, as mentioned above.
  - **Risk:benefit assessment**. In cases where the therapeutic window of an IND is narrow, and if the adverse events are clearly linked to the mechanism of action of the drug, then QSP simulations could help to estimate the risk:benefit ratio for patients, or to tease out for which subpopulations this could be minimized.
  - **Relative value within the portfolio.** Especially in big pharmaceutical companies, different programs compete for the funding of their CDPs, sometimes even for the same pool of patient population. QSP simulations of the studies included in those CDPs can help identify the assets or studies to accelerate or to focus most resources on, based on the estimated level of clinical outcome.
  - **Differentiation from competitors / standard of care.** If there is enough pharmacological data available on approved SoC or on INDs competing against a company’s proprietary asset, then QSP models can provide a sandpit where the success of differentiation strategies (e.g., alternative routes of administration, schedules, doses, formulation, target population, etc.) can be tested.

- **Regulatory submissions**: as described by Bai et al. [12], QSP-based evidence can help support regulatory submissions regarding Investigational New Drugs (INDs), New Drug Applications (NDAs), Biologics License Applications (BLAs), Investigator’s Brochures (IBs), Briefing Books (BBs), pediatric study plans (PIPs, iPSPs, PSPs), answers to regulatory questions (RTQs), etc., in several aspects:
  - **Dose justification.** This applies to phase 1 dose-finding/-escalating, recommended phase 2 dose(s) for dose ranging studies (RP2D), and recommended phase 3 dose (RP3D) selection, including pediatric subpopulations.
  - **Label recommendations.** This refers to supporting the inclusion or exclusion of certain patient subpopulations to address regulatory safety concerns.
  - **Post-Marketing Requirements (PMRs) and Commitments (PMCs) Efficacy/Safety supplements**. When a certain additional study is required by regulators as a condition for drug approval, a QSP analysis could support the waiving of such study. This includes cases where the dose for a new disease indication is bridged to an existing approved indication.

### Empirical vs. QSP models

When it comes to the way in which models are embedded into the decision-making processes, there are stark differences between QSP and the more traditional, empirical pharmacometrics methods. Table 1 summarizes the key characteristics that lead to contrasting frames of mind when adopting each of these types of methods as MIDD/MID3 tools.

**Table 1:** Comparison of the typical major characteristics of QSP versus empirical pharmacometric projects in industry, when used as MIDD/MID3 tools

	Quantitative and Systems Pharmacology	Classic Empirical Pharmacometrics
Premise	All empirical observations (biological and clinical) are connected at a multiscale level	There is an optimal system of equations that can fit to and describe empirical observations
Motivation	Target-centric or disease-centric	Drug-centric
Pipeline phase	Target ID to Life Cycle Management	Lead Optimization to Life Cycle Management
Trigger	Strategic decision or question for the project team	Clinical data availability
Timelines	Months	Weeks
Context of use	Flexible / Adaptable extrapolation	Limited / Restricted extrapolation
Assumptions	Transparent and explicit / Often incomplete	Blind or undeclared / Often implicit
Basis for assumptions	First principles and data	Data
Types of input data	In-vitro and clinical (occasionally in-vivo)	Clinical/In-vivo (occasionally in-vitro)
Repurposing	Possible/Likely	Rare/Impossible
Validation	Unusual for Discovery, and likely for Clinical Development/Prior to decision-making	Unusual/Post-decision making

The premise or key starting point for QSP modelers is to describe the connectivity between observed events, specifically between biology and clinical (less often, in-vivo) endpoints. The big advantage of this is that project teams can start to build QSP models well before they generate clinical (or in-vivo) data, just bearing in mind the timing when the MIDD/MID3 decision(s) need to be informed. The flipside is that QSP models take a long time to build, because the integration of biological and clinical information requires a high level of complexity and deep interactions with the Discovery Research and the Clinical Development subject-matter experts (SMEs). For this reason, it is very important to plan for the need to QSP models well ahead of the decision point, in the order of 4 months to 1 year in advance. The actual time it takes to develop a QSP model will depend on the amount and quality of the data available for its calibration, and on how challenging the repurposing of previous QSP models can be [28].

In contrast, empirical pharmacometrics models require shorter timelines than QSP models, in the order of 1 to a few weeks. This is because pharmacometrics seeks to find an optimal system of equations that can describe accurately a single set of in-vivo or clinical data. The planning of an empirical model or analysis is tied to the dates of data readout, which are generally close an MIDD/MID3 decision point. The data that pharmacometricians use tend to be from trials that follow a design as close as possible to the trial they intend to simulate and inform, in terms of the drug, the disease characteristics and patient demographics. This limits the applicability of empirical models to the same context in which the data was generated, making their repurposing to inform an alternative drug program in the pipeline very rare. In contrast, QSP models can be informed with clinical data above study level, even beyond asset level, as long as the target or the mechanism of action of the drug or the indication correspond to the context of the decision to be informed. This gives QSP models a span of applicability to a wider context than for empirical models, meaning that repurposing and extrapolation with QSP models to new drug projects in the pipeline is easier and more common than with empirical models.

Regarding qualification, the requirement for model validation with an external dataset is dependent on the type of model and on the drug development phase. Generally, during Discovery QSP models are used to generate hypotheses, so models are used unqualified until experimental data is generated [29]. As models evolve into later drug development phases, and given the existence of several ‘moving parts’ in a QSP model [30], it is good practice to validate the model performance with an external clinical dataset prior to using it for prospective simulations meant to inform clinical development decisions (see section about Technical validation vs. Credibility and risk in a context of use). These datasets may be obtained from standard of care (SoC) treatments (in precedented mechanisms) or from competitor trials with INDs. However, such data are difficult or impossible to obtain, in which case it is very important to inform the decision-makers of the limitations of the prospective QSP simulations, and provide them with some measure of confidence in the predictions. This is often the case when First Time in Human (FTIH) studies are informed. In contrast, empirical pharmacometric models tend to be validated with clinical data after prospective simulations have been produced for MIDD/MID3 decision-making, and the new study readouts become available. Then the new data is normally added to the original training dataset and used to re-calibrate the model.

### Fit-for-purpose vs. platform models

Broadly speaking, empirical pharmacometrics models are considered ‘fit-for-purpose’, whilst QSP models are deemed ‘complex’. In reality, there are multiple degrees of complexity and granularity within each type of approach, leading to concepts like mechanistic empirical models, minimal PBPK models, and more recently platform QSP models.

A platform model is a ‘framework’ that interlinks biological processes, biomarkers, and clinical endpoints in a specific disease indication, trying to capture as much biological complexity as possible without the bias of developing pathways around specific molecular drug targets. Because of this lack of initial bias, these models are expected to enable the emergence of unknown unknowns about the system behavior. Their main advantage is that they can be repurposed multiple times for new drug development projects, by embedding ad-hoc the specific drug target relationship to the biological processes already described in the disease framework. However, this leads to an organic growth of the platform that naturally increases the simulation processing time, requires constant updating of the model structure and parameterization, and hence can add an unsustainable overhead for Pharma companies. From this point of view, probably a symbiosis between CROs/academia and industry to develop these platform models would be ideal: the former possess the agility and capability necessary to maintain, expand and apply these platform models at scale, whilst the latter can provide a meaningful context, subject matter expertise, and drug-specific data. For this to become a reality, it would be important to embed an efficient knowledge management process and intentional two-way communication between partners. Examples of platform models can be found in the literature, focused on immuno-oncology [31], ACDs (antibody-drug conjugates) [32], lipoprotein metabolism [33] or Alzheimer’s Disease [34], to cite a few areas.

The alternative to platform QSP models would be ‘fit-for-purpose’ QSP models. These are models developed with one or more selected drug target(s), with the aim to describe only the cellular mechanisms that link that specific target (or set of targets) to a set of clinical biomarkers in the context of a pre-determined indication. These models are more cost-effective than platform models because they require less resources (data, time, and modelers) for building and maintenance, but can be more challenging when more drug targets need to be added if the model building process is documented insufficiently. They may also miss emerging behaviors that platform models would include by design, thus missing the opportunity to identify unknown unknowns in the mechanism of action of the investigational drug. Most QSP models in the public domain are fit-for-purpose.

A concept that has evolved within Pharma and gained traction with the development of platform models is that of ‘virtual patients’. These are in silico representations of individual subjects (healthy volunteers or patients), each one defined by a fixed set of values for their baseline characteristics and/or QSP model parameters. Those values are taken from a distribution within expected ranges (known or hypothesized) and need to lead to a ‘plausible’ emerging behavior, which means that the longitudinal biomarker simulations for the virtual patient fall within the margins observed in real subjects. When trying to estimate or reproduce the results of a specific clinical study, then the patient prevalence in the model should match that of the trial, i.e., the averages and distributions of their disease characteristics should be the same. Several methods to develop QSP virtual patients and cohorts have been discussed in the literature, together with ways to optimize the challenging computational cost that these approaches represent [35–44]. The estimation of clinical responses of a single patient has led to the idea of ‘Virtual Twins’ in PBPK modelling [24], akin to ‘Digital Twins’ in the medical devices space. Translated to the QSP space, and combined with -omics analyses and Machine Learning (ML) methods [45], this could potentially open the door to Personalized Medicine, with truly individualized and adaptive drug treatments for patients.

### Technical validation vs. credibility and risk in a context of use

Given the complexity inherent to the process of developing QSP models, the qualification methods used for more traditional MIDD/MID3 approaches are not sufficient. Therefore, numerous approaches have been proposed by the QSP community in order to guarantee, address and communicate the quality of QSP models in general [1, 13, 30, 46–48].

From an industry perspective, all the recommendations are very valuable, but perhaps the most relevant point of view is that coming from regulatory agencies [48]. The role of regulatory reviewers is to ensure that the QSP evidence provided by sponsors is scientifically sound and valid. Therefore, the technical soundness and ability of the model to adequately simulate real-scenario situations (‘qualification’ for the EMA, and ‘verification & validation’ (‘V&V’) for the FDA) is important. However, they are not sufficient because ultimately, regulators base their decisions on an assessment of the associated risk:benefit for patients and public health. This means that the QSP model and derived simulations need to be valid for the specific decision that they are supporting, and in addition sponsors need to lay out the consequences for patients, should the model underperform.

In this regard, two recent papers have been issued independently by the EMA [49] and the FDA [50] to propose model credibility frameworks (‘Risk-informed evaluation framework’ and ‘Risk-informed credibility assessment framework’, respectively), both inspired in the ASME V&V40 framework for medical devices [51]. There are five central concepts that stand out from both proposals, all of which provide a link between the technical validation of the models and the decision-making process:

- **Context of use (CoU).** This is a statement that defines the specific role and scope of the model used to address the question/decision of interest. According to the EMA, the CoU is a critical reference point for the regulatory evaluation of any qualification application, and is considered to be the full, clear and concise description of the way in which the methodology is to be used and of the purpose of the use [52].
- **Regulatory impact / Model influence.** This is the degree of importance of the simulations in the decision-making process when considering all available evidence. It can be low (the simulations only describe evidentiary clinical and/or non-clinical data coming from other sources), high (the simulations are a key source of evidence, e.g. replacing data from a clinical trial) or medium (the simulations complement other evidence, e.g. to support dose selection in a particular patient sub-population where some clinical data is available).
- **Decision consequence.** This reflects the magnitude of the consequence of an adverse outcome resulting from an incorrect decision based on the model. It can be low (an incorrect decision would not result in adverse outcomes in patient safety or efficacy), high (an incorrect decision could result in severe adverse outcomes in patient safety or efficacy) or medium (an incorrect decision could result in minor to moderate adverse outcomes in patient safety or efficacy).
- **Model risk**. The ‘Model influence’/‘Regulatory impact’ and the ‘Decision consequence’ ratings are combined into an overall ‘Model risk’ ranking for the given CoU. This level of risk is then used to plan the technical soundness activities and acceptability criteria (i.e., ‘qualification’ or ‘V&V’) that should be used to assess the credibility of the model.
- **Credibility assessment.** An assessment of whether there is sufficient model credibility to support the CoU can be made by evaluating the applicability of the performed V&V activities to the CoU, mindful of the ‘Model risk’.

The EMA paper proposes to use this framework to evaluate any type of mechanistic in silico models (including QSP), and the FDA paper applies this to PBPK models to initiate a debate about its applicability to MIDD/MID3 approaches in general. At the moment, though, there are no clear, specific guidelines from either agency for the submission of QSP-based evidence.

## Conclusions/Summary

In the last 10 years, QSP has evolved from infancy to adulthood in industry, from featuring as a unique example of regulatory submission to becoming a recognized tool in the MIDD/MID3 ensuite.

Its application in industry started in Discovery Research projects, and markers of activity such as the volume of publications and of regulatory submissions indicate that its use has extended successfully into the Clinical Development arena. This is still work in progress, and not without challenges, such as a lack of understanding of QSP’s RoI by senior leadership, a confirmatory mindset of clinical teams, rigid operational environments, and a lack of standardization for QSP in regulatory submissions.

QSP may be the only MIDD/MID3 approach capable of creating a continuum of knowledge between the earliest phase in Discovery and the latest stage in Clinical Development, from Target Identification to Life Cycle Management. The mindset for QSP model development shows significant differences from that required for empirical pharmacometric models. However, rather than compete against other MIDD/MID3 tools, QSP needs to play to its strengths, capitalize on the unique value that it can add to pharma industry decision-making. Finding an adequate balance between all types of MIDD/MID3 approaches (hopefully including Machine Learning, in a not-too-distant future) can only lead to win-win situations.

In addition, if we want to see the impact of QSP in industry continue to increase, then the new generations of modelers, pharmacometricians and clinical pharmacologists in academia, industry, CROs and regulatory agencies need to fully understand the context of use for QSP approaches – hopefully this article will help towards that.
